# A new species of the millipede genus *Cryptocorypha* Attems, 1907, from northern Thailand (Polydesmida, Pyrgodesmidae)

**DOI:** 10.3897/zookeys.833.32413

**Published:** 2019-04-01

**Authors:** Natdanai Likhitrakarn, Sergei I. Golovatch, Ruttapon Srisonchai, Chirasak Sutcharit, Somsak Panha

**Affiliations:** 1 Division of Plant Protection, Faculty of Agricultural Production, Maejo University, Chiang Mai 50290, Thailand Maejo University Chiang Mai Thailand; 2 A.N. Severtsov Institute for Problems of Ecology and Evolution, Russian Academy of Sciences, Leninsky pr. 33, Moscow 119071, Russia A.N. Severtsov Institute for Problems of Ecology and Evolution, Russian Academy of Sciences Moscow Russia; 3 Animal Systematics Research Unit, Department of Biology, Faculty of Science, Chulalongkorn University, Bangkok, 10330, Thailand Chulalongkorn University Bangkok Thailand

**Keywords:** Chiang Mai, Diplopoda, Henrik Enghoff, Huai Hong Khrai Royal Development Study Centre

## Abstract

The millipede family Pyrgodesmidae and the genus *Cryptocorypha* are recorded from Thailand for the first time, being represented there by *C.enghoffi***sp. n.** The new species is distinguished by the evident apicodorsal trichostele on the last tibia of both sexes and the gonopodal telopodite being particularly complex, quadripartite, consisting of the longest, mesal, suberect solenomere branch; a slightly shorter, similarly slender, acuminate endomere branch tightly appressed to the solenomere; a somewhat shorter, caudal, strongly curved, armed exomere process; and a very distinct, low, lateral, sac-shaped velum at their base. This situation strongly resembles the one observed in the geographically closest *C.perplexa* Golovatch & VandenSpiegel, 2015, from Myanmar, but the shapes and armament of all outgrowths of the gonopodal telopodite are clearly different. A key to all three *Cryptocorypha* pecies known from Indochina or Myanmar and an updated checklist of all 21 species of the genus are provided.

## Introduction

The genus *Cryptocorypha* Attems, 1907, is one of the few relatively speciose genera of the mainly tropical millipede family Pyrgodesmidae which is among the largest in the entire class Diplopoda. The family Pyrgodesmidae currently comprises more than 170 genera and nearly 400 species (Minelli 2015, Golovatch et al. 2017). *Cryptocorypha* has recently been reviewed, rediagnosed (Golovatch et al. 2011b, 2013, 2017, Golovatch and VandenSpiegel 2015), and shown to encompass 20 species ranging from central and eastern tropical Africa, through India, Sri Lanka and Myanmar, to East Asia, southern China, Indochina, western Indonesia, and even Melanesia (Table 1).

Most of the congeners tend to show very narrow distributions, with only a single species, *C.ornata* (Attems, 1938), being extremely widespread on tropical islands and archipelagos in the Indian and Pacific oceans, apparently due to anthropo- and/or ornithochory (Minelli 2015, Golovatch et al. 2017).

The present paper puts on record a new species of this genus, the first to be found in Thailand. An updated checklist of all 21 species of *Cryptocorypha* known to date and a key to all three congeners from Indochina or Myanmar are also provided.

**Table 1.** Described *Cryptocorypha* arranged in alphabetic order and supplied with geographical details.

**No.**	**Species**	**Locality or localities**
1	*C.areata* (Carl, 1932)	India, Upper Palnis, Kodaikanal and environs, 2,200 m; Maryian-shola, 2,300 m; Kukkal-shola, 1,900 m; near Pumberai, 1,900 m; Lower Palnis, Thandikudi, 1,500 m; Travancore, between Palni and Anaimala Hills, 1,850 m (Carl 1932)
2	*C.bocal* Golovatch, Nzoko Fiemapong & VandenSpiegel, 2017	Congo D.R., South Kivu Province, Itombwe, Uvira District, road-km 10 from Katobo to Kahololo, 03°12'S, 28°51'E, 2,400–2,800 m (Golovatch et al. 2017)
3	*C.chernovi* Golovatch, Geoffroy & VandenSpiegel, 2013	Vanuatu, Espiritu Santo Island, Rotal, near Rotal hole, 15°15'10.1"S 167°03'30.5"E, 250 m; Boutmas, near the entrance to Fapon Cave, 15°19'51.7"S 166°57'53.6"E, 380 m; Malo Island off Espiritu Santo, Avorani, 15°42'22.1"S 167°07'43.5"E, 110 m (Golovatch et al. 2013)
4	*C.diffusa* (Brolemann, 1920)	East Africa, Mt. Kilimanjaro, a small series near a forest, 2,700–2,800 m (Brolemann 1920); Kenya, Taita Hills, Mbololo Forest, 03°19'S, 38°27'E, 1,800–1,900 m; Yale Forest, 03°39'S, 38°33'E; Fururu Forest, 03°26'S, 38°20'E; Ngangao Forest, 03°22'S, 38°21'E; Saga Forest, 03°50'S, 38°58'E; Mwachora Forest, 03°24'S, 38°22'E (Golovatch and VandenSpiegel 2014); Mission Zoolg. I.R.S.A.C en Afrique Orientale, Tanganyika terr. (= Tanzania), Ngorongoro, Bocagere Region, 2,300 m; Mt. Oldeani versant Est, mountain forest with *Bambusa*, 2,350–1,950 m; mountain forest, 1,880–1,950 m; Mt. Oldeani versant N.O, etrepage sous *Hagenia*, 2,600 m (Golovatch et al. 2017)
5	*C.dimorpha* Golovatch, Nzoko Fiemapong & VandenSpiegel, 2017	Congo D.R., Kivu, Maniema Province, Mwenga, 03°03'S, 28°26'E (Golovatch et al. 2017)
6	*C.enghoffi* sp. n.	Thailand, Chiang Mai Province, Doi Saket District, Huai Hong Khrai Royal Development Study Centre, 18°52'47"N, 99°13'22"E, 445 m
7	*C.hoffmani* Golovatch, Semenyuk, VandenSpiegel & Anichkin, 2011	Vietnam, Dong nai Province, Nam Cat Tien National Park, ca. 150 m (Golovatch et al. 2011a, 2011b)
8	*C.japonica* (Miyosi, 1957)	Japan, Tokyo, Futako Tamagawa (Miyosi 1957)
9	*C.kandyana* (Carl, 1932)	Sri Lanka (Ceylon), Kandy (Carl 1932)
10	*C.kumamotensis* (Murakami, 1966)	Japan, Ehime Prefecture, Niihama, Oshima; Iyo-Mishima, Kinsha (Murakami 1966)
11	*C.leia* Chamberlin, 1945	Indonesia, Java, Goenong Malabar, 1,600 m (Chamberlin 1945)
12	*C.leleupi* Golovatch, Nzoko Fiemapong & VandenSpiegel, 2017	Congo D.R., South Kivu Province, Itombwe, Uvira District, road-km 10 from Katobo to Kahololo, 03°12'S, 28°51'E, 2,800 m (Golovatch et al. 2017)
13	*C.monomorpha* Golovatch, Nzoko Fiemapong & VandenSpiegel, 2017	Congo D.R., Kivu, Dorsale de Lubero, Mt Muleke, versant Sud, village Itala, 00°17'S, 29°15'E, 1,820 m (Golovatch et al. 2017)
14	*C.nympha* Loksa, 1967	Republic of the Congo (Congo-Brazzaville), ORSTOM-Park (Loksa 1967)
15	*C.ornata* (Attems, 1938)	Nearly pantropical anthropo- and/or ornithochore species (Adis et al. 1998; Golovatch et al. 2017). Hawaiis (Attems 1938, 1940); Saint Helena Island (Hoffman 1977); Cook Islands; Marquesas Islands; Tahiti Island; Hong Kong (Adis et al. 1998); Taiwan (Golovatch et al. 2011a)

## Materials and methods

The specimens were hand-collected from Huai Hong Khrai Royal Development Study Centre during the rainy season (during the months of April to October in 2015 and 2016). Live animals were photographed in their habitats and then taken for photography in the laboratory using a Canon 70D digital camera with a Canon EF-S 60mm f/2.8 Macro USM lens. After that, the specimens were preserved in 75% ethanol. The morphological characters were studied in the laboratory using uncleaned specimens and an Olympus stereo microscope. The terminology used follows that accepted in the most recent publications (Golovatch et al. 2011b, 2013, 2017, Golovatch and VandenSpiegel 2015). Scanning electron micrographs (SEM) were taken with a JEOL, JSM–5410 LV microscope with gold coating, and the material returned from stubs to alcohol after examination. Images of the holotype habitus were taken in the laboratory and assembled using the “CellD” automontage software of the Olympus Soft Imaging Solution package and the gonopods of a paratype were dissected and illustrated under Euromex iScope microscopes. The holotype and most of the paratypes are housed in the Museum of Zoology, Chulalongkorn University (CUMZ), Bangkok, Thailand. A few paratypes have also been donated to the collections of the Zoological Museum, State University of Moscow, Russia (ZMUM), Natural History Museum of Denmark, University of Copenhagen, Denmark (ZMUC), Naturhistorisches Museum Wien, Austria (NHMW), and Natural History Museum, London, Great Britain (NHML), as indicated in the text.

The Animal Care and Use Protocol Review No. 1723018 was applied.

The geographical coordinates and elevation were recorded by means of a Garmin GPSMAP 60 CSx using the WGS84 datum and subsequently double-checked with Google Earth.

## Taxonomic part

### Family Pyrgodesmidae Silvestri, 1896

#### Genus Cryptocorypha

Taxon classificationAnimaliaPolydesmidaPyrgodesmidae

Attems, 1907

##### Diagnosis.

The genus is characterized within Pyrgodesmidae by an unusually flat body with 19 or 20 segments (either in both sexes or 19 solely in the male) and only a slightly convex dorsum, coupled with 6+6 faint lobulations or 11 radii at a regularly rounded anterior margin of a flabellate collum that fully covers the head from above; usually three or four (rarely five) more distinct lobulations at the lateral margins of poreless and pore-bearing paraterga, respectively; a normal pore formula (5, 7, 9, 10, 12, 13, 15–18(19)) with the ozopores not borne on porosteles, but opening flush on the dorsal surface at the base of the penultimate lobulation; the absence of anterior and the presence of only very few (1–2) caudal lobulations; the development of 2–3 transverse, often irregular rows of small and non-differentiated knobs/tuberculations on each postcollum metatergum; and a dorsally fully exposed epiproct. The last tibia in the male or even in both sexes is often, but not always, with a conspicuous, long, setigerous, apicodorsal cylinder (= trichostele). The gonopods are with relatively small coxae and a shallow gonocoel that leaves the telopodites very strongly exposed and in situ held (sub)parallel to each other; each telopodite is 2-, 3- or 4-partite, with a strongly developed, slender, often fimbriate, mesal solenomere branch (usually the longest) and a typically sac-shaped velum at its base, sometimes also with 1–2 adjacent processes (exo- and/or endomere, depending on position) (Golovatch et al. 2017).

#### Cryptocorypha enghoffi sp. n.

Taxon classificationAnimaliaPolydesmidaPyrgodesmidae

http://zoobank.org/D2E1D3D0-3968-41B0-AD60-7D610F34F832

Figs 1 , 2 , 3 , 4

##### Holotype.

♂ (CUMZ), Thailand, Chiang Mai Province, Doi Saket District, Huai Hong Khrai Royal Development Study Centre, 445 m a.s.l., 18°52'47"N, 99°13'22"E, 06/05/2015, leg. N. Likhitrakarn. **Paratypes.** 2 ♂, 3 ♀, 1 subadult (19 segments), 1 juvenile (18 segments) (CUMZ), 1 ♂, 1 ♀ (ZMUM), same locality, together with holotype. 1 ♂, 1 ♀, 2 subadult (19 segments) (CUMZ), 1 ♂, 1 ♀ (ZMUC), 1 ♂, 1 ♀ (NHMW), 1 ♂, 1 ♀ (NHML), same locality, 09/06/2016, leg. N. Likhitrakarn.

##### Name.

Honours Henrik Enghoff, a globally renowned specialist in Diplopoda and one of the pioneers of diplopodological research in Thailand.

##### Diagnosis.

Differs from other species of the genus by the presence of 20 body segments in both sexes, coupled with an evident apicodorsal trichostele on the last tibia of both sexes (Fig. 4F) and in the gonopod structure being particularly complex, similar to that of *C.perplexa* Golovatch & VandenSpiegel, 2015, but differs clearly in the shapes and armament of all four main outgrowths of the telopodite (Fig. 4A–D).

##### Description.

Length ca. 12.1 mm, width of midbody segments 2.95 and 1.55 mm on pro- and metazonae, respectively (holotype). Length of adults ca. 11.5–12.8 mm (♂ paratypes) and 14.5–15.2 mm (♀ paratypes), width of midbody pro- and metazonae 0.8–1.2 and 2.2–2.6 mm (♂ paratypes) or 1.2–1.8 and 2.8–3.4 mm (♀ paratypes), respectively.

**Figure 1.**
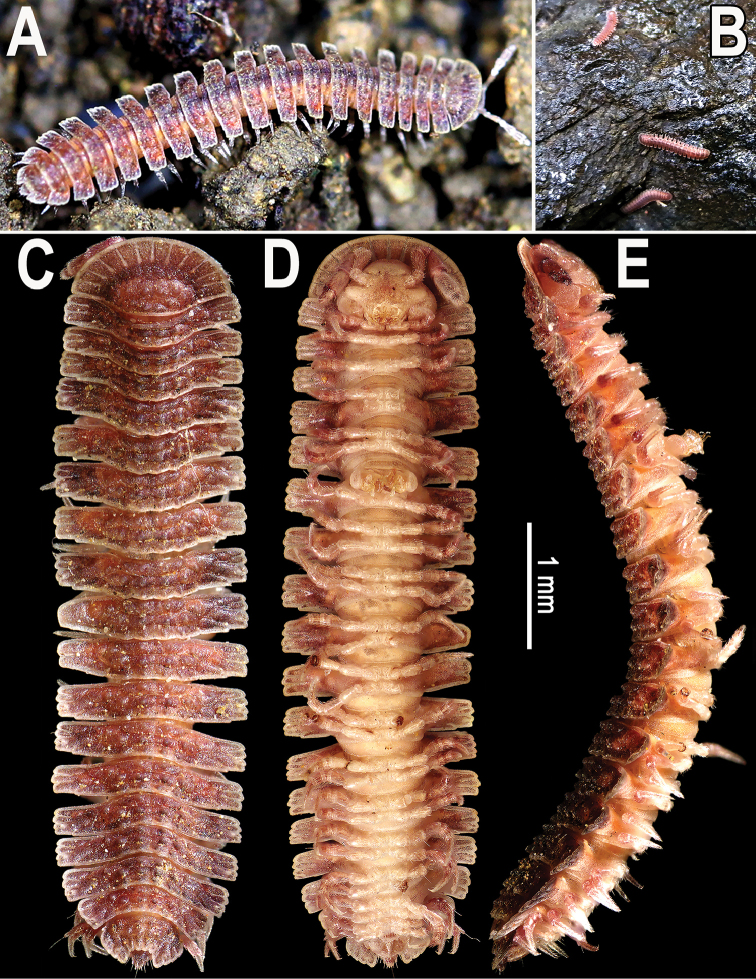
*Cryptocoryphaenghoffi* sp. n., **A** ♀ paratype **B** a few paratypes **C, D** holotype **A, B** habitus, live coloration in their habitat **C–E** habitus and coloration in alcohol, dorsal, ventral and lateral views, respectively.

Coloration of live animals uniformly reddish to purplish red (Fig. 1A, B), antennae, legs, and venter mainly lighter, yellowish to reddish (Fig. 1A); coloration in alcohol, after three years of preservation, faded to reddish (Fig. 1C–E) or light brown, antennae and legs light red to light brown, while venter yellowish to nearly pallid (Fig. 1D, E).

**Figure 2.**
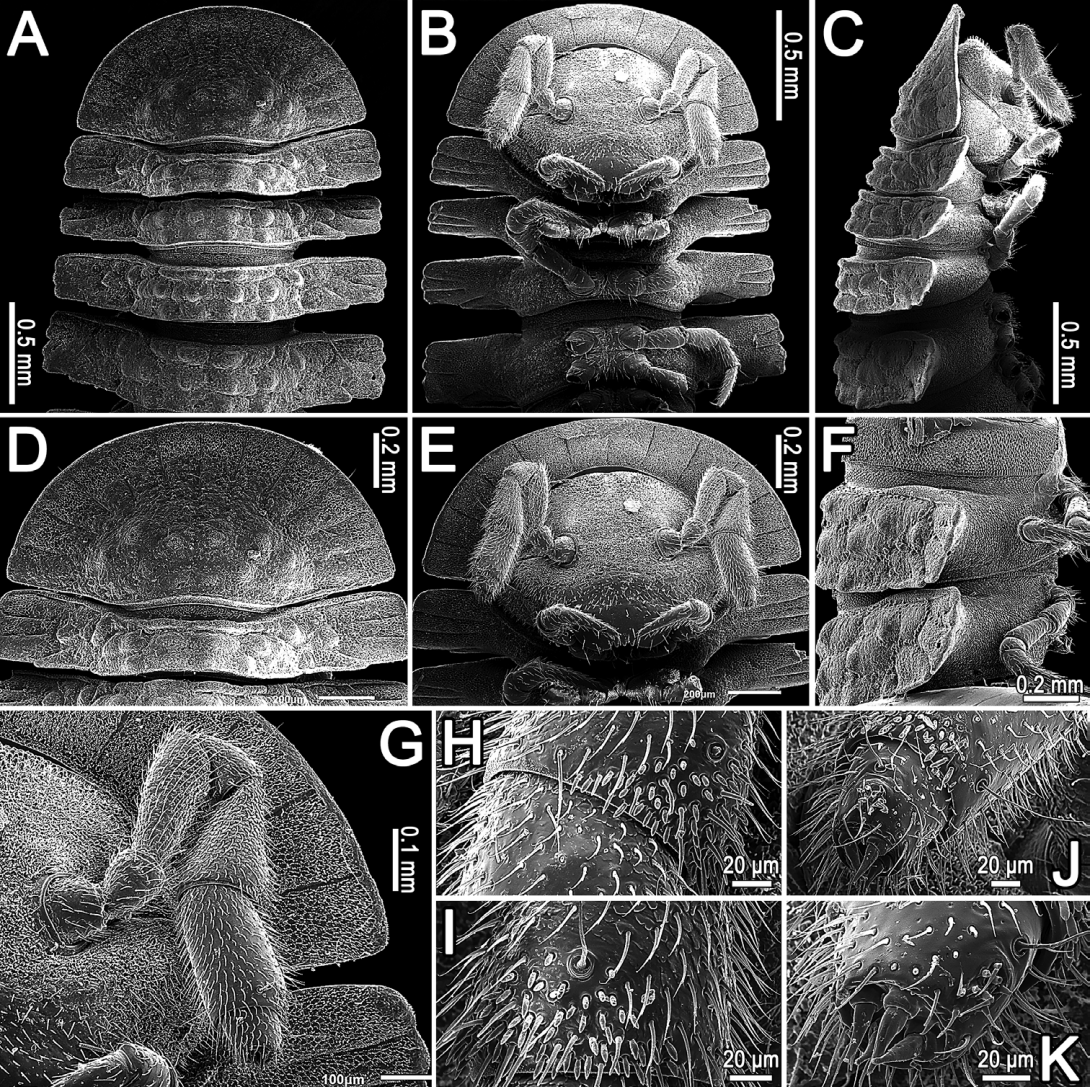
*Cryptocoryphaenghoffi* sp. n., ♂ paratype. **A–C** anterior part of body, dorsal, ventral and lateral views, respectively **D** collum, dorsal view **E** head, ventral view **F** segments 8, 9, lateral view **G** antenna, ventral view **H–K** right antenna **H** bacilliform sensilla on antennomere 5, sublateral view **J, I** bacilliform sensilla on antennomere 6, subventral and sublateral views, respectively **K** tip of right antenna, sublateral view.

**Figure 3.**
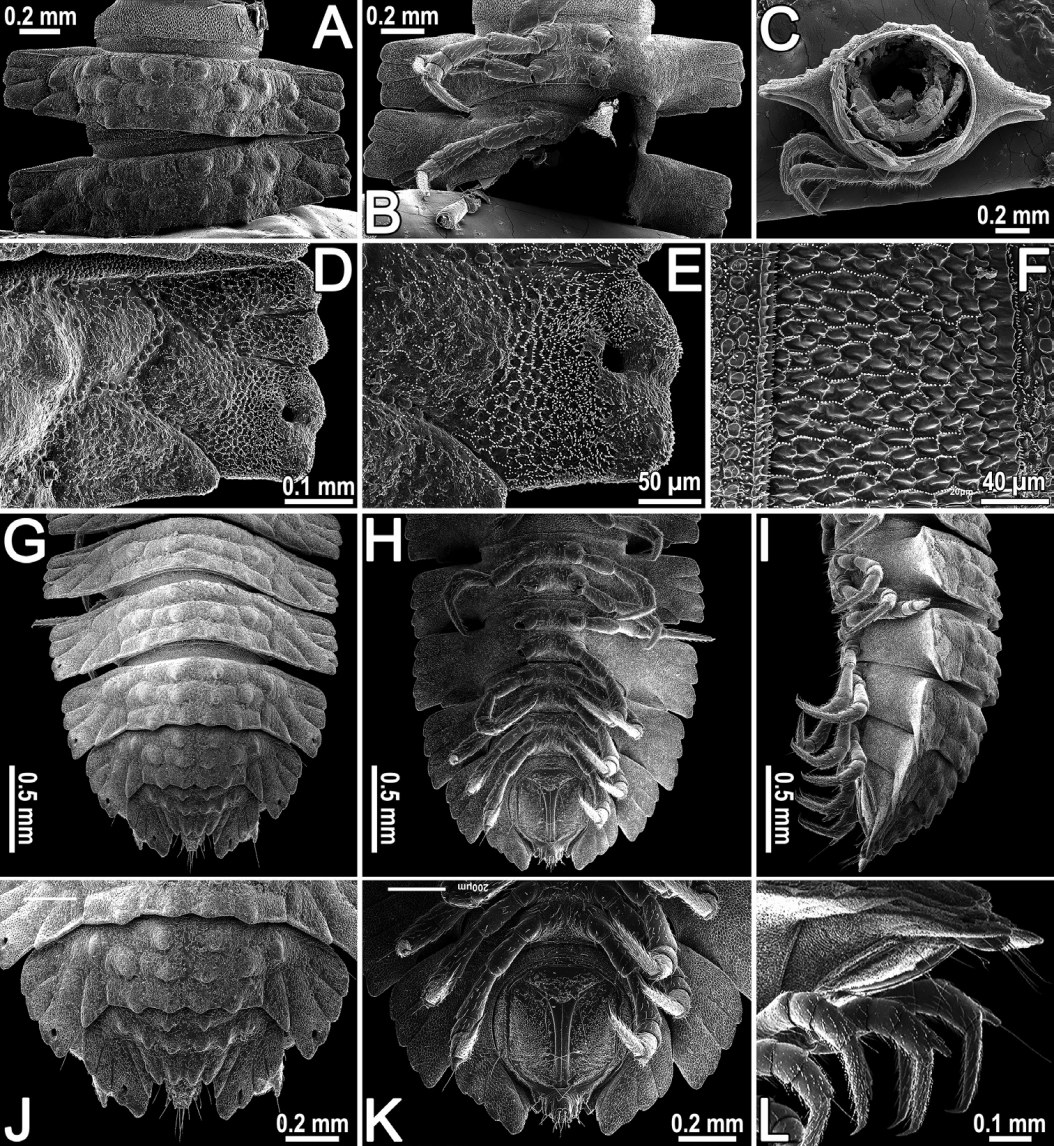
*Cryptocoryphaenghoffi* sp. n., ♂ paratype. **A**, **B** segments 8, 9, dorsal and ventral views, respectively **C** cross-section of segment 8, caudal view **D** paraterga of segment 9, dorsal view **E** poriferous paratergum of segment 9 **F** tegument texture in the region of a stricture between pro- and metazonae, dorsal view **G–L** posterior part of body, dorsal, ventral, lateral, dorsal, ventral and lateral views, respectively.

Body robust, with 20 segments (♂, ♀). Pro- to metazonum width ratio close to 1:2. In width, head << collum < segment 3 = 4 < 2 < 5 < 6–14(15) (♂, ♀), thereafter body rapidly tapering towards telson (Figs 1C, D, 3G, H). Head subovoid (Fig. 2B, C, E), slightly transverse, densely setose in clypeolabral region, micropapillate; epicranial suture superficial. Interantennal isthmus approximately twice as large as either diameter of antennal socket or antennomere 1 (Fig. 2B, E).

Antennae short and clavate (Figs 1A, D, 2B, C, E, G), in situ reaching body segment 3 (♂, ♀) when stretched laterally or ventrolaterally; in length, antennomere 1 < 2 < 4 <7 < 3 < 5 < 6; antennomeres 5–7 each with a more or less compact apicodorsal group of bacilliform sensilla (Fig. 2G–K).

Collum flabellate (Figs 1A, C, 2A–E), completely covering the head from above, anterior margin regularly rounded, with 11 equal, long and evident radii (Figs 1C, 2A); middle and caudal parts with two transverse, arched, rather faint rows of low bosses (Figs 1C, 2A, C, D). Paraterga set at approximately upper 1/3 (♂, ♀) of body height, subhorizontal to faintly declivous (♂, ♀) (Figs 1E, 2C). Dorsum moderately convex, its outline smoothly extending onto paraterga (Fig. 2C).

Tegument encrusted with a microspiculate cerotegument, dull, beset with microvilli (Figs 2A, C, D, F, 3A, C–G, I, J). Prozonae and strictures between pro- and metazonae very delicately microgranulate, also beset with microvilli (Fig. 3F), conforming to the pattern observed in *C.ornata* and several other genera and species of Pyrgodesmidae (cf. Akkari and Enghoff 2011). Metaterga with three transverse rows of non-differentiated tuberculations and distinct rows of usually transversely oblong, polygonal to rounded, low bosses (Figs 2A, 3A, J), except for collum and segments 2–4 showing two transverse rows of such tuberculations (Fig. 2A, D), each of the latter typically surmounted by minute, setigerous, spherical knobs (Fig. 3D). Paraterga areolate-rugose, beset with microvilli arranged in a polygonal alveolate pattern (Fig. 3E; see also Akkari and Enghoff 2011 for comparison). Tergal setae mostly abraded, retained ones inconspicuous and very short.

Postcollum paraterga very broad, thin and slightly, but clearly lobulate laterally (Figs 1A, C, 2A, B, 3A, B, D, G, H, J), with three lobulations in all poreless segments, four lobulations in all pore-bearing ones, all also delimited by very long, rather evident radii both dorsally and ventrally; anterior marginals absent, but two caudal marginals evident.

Pore formula normal: 5, 7, 9, 10, 12, 13, 15–19, ozopores being very small, round, discernible dorsally at base of 3^rd^ lobulation (Figs 2A, F, 3A, D, E, G, J).

Limbus microspiculate, each caudal crenulation being very finely and sharply spinulose (Fig. 3F).

Epiproct readily visible from above, not hidden under 19^th^ segment (Figs 1A, 3G, J), with four strong setae on top (Fig. 3K).

Hypoproct subtriangular, caudal edge with 1+1 strong and widely separated setae on evident knobs (Fig. 3K).

Sterna wide, approximately twice as broad as diameter of coxal socket (Figs 1D, 2B, 3B, H, K), moderately setose, without modifications, superficially impressed along main axis. Epigynal ridge behind ♀ legs 2 low and inconspicuous. Gonopod aperture transversely oblong-oval, caudal and lateral margins thin and slightly elevated.

**Figure 4.**
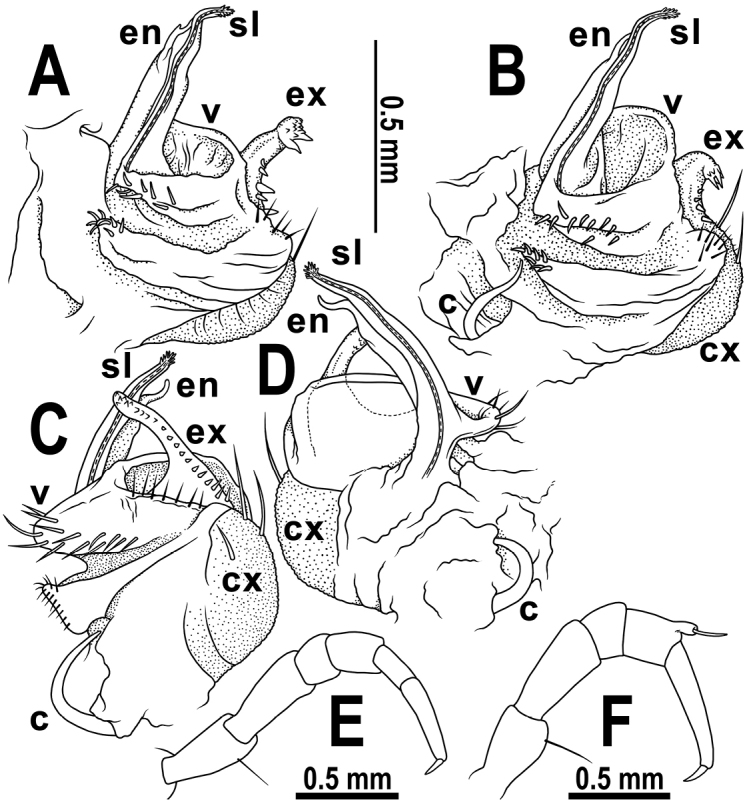
*Cryptocoryphaenghoffi* sp. n., ♂ paratype. **A–D** left gonopod, sublateral, submesal, suboral, lateral and mesal views, respectively **E** midbody leg, lateral view **F** last leg, lateral view. Abbreviations: **c**, cannula **cx**, coxite **en**, endomere **ex**, exomere **sl**, solenomere **v**, velum.

Legs long and slender (Fig. 4E), longer than width of paraterga, densely setose, last tibiae with evident apicodorsal trichosteles in both sexes (Figs 3I, K, L, 4F); in length, tarsi > femora > prefemora >> tibiae > coxae > postfemora (♂, ♀), neither adenostyles nor tarsal brushes. Claws simple, slightly curved ventrad.

Gonopods (Fig. 4A–D) very complex, in situ held parallel to each other; coxite rather small, boat-shaped, gonocoel shallow, cannula simple. Each telopodite grossly quadripartite: (1) an evident, long, suberect, rod-shaped, apically unequally bifid and acuminate endomere tightly appressed to and starting at base of (2) the longest, suberect, rod-shaped, distally curved, apically conspicuously and densely fringed/fimbriate solenomere, followed first (3) by a sac-shaped, mesally irregularly membranous, low velum and then (4) by a conspicuous, long, clearly papillate/dentate, strongly curved, apically slightly clavate and rounded exomere .

##### Remarks.

This new species was found walking on a rock surface (Fig. 1B). The air was very humid, this being characteristic of the rainy season. The specimens were found in the Dry Dipterocarp Forest at the Huai Hong Khrai Royal Development Study Centre. This study centre was established under the royal initiative in 1982 in the area of Khun Mae Kuang National Forest Reserve, Chiang Mai Province for conducting research and experimentation using appropriate progressive methods which suited the development needs of the Northern Region, especially the conservation of watersheds, reforestation and agricultural development. It covers approximately 8,500 rai (1,360 hectares).

### Key to the species of *Cryptocorypha* currently known to occur in Indochina or Myanmar, chiefly based on ♂ characters

**Table:** 

1	Body larger, 10–15.2 mm long. Gonopods complex, telopodite clearly quadripartite (Fig. 4A–D)	**2**
–	Body smaller, 4.0–4.5 mm long. Gonopods simple, telopodite bipartite, with only an evident solenomere branch protruding above a hypertrophied sac-shaped velum (Golovatch et al. 2011b: figs 39–44). Vietnam	*** C. hoffmani ***
2	Body smaller, 10–11 mm long, width of midbody metazonae 1.9–2.0 mm. Velum shorter and smaller, exomere suberect, nearly as long as endomere, with an evident stump-shaped outgrowth caudally at base (Golovatch and Vandenspiegel 2015: figs 3C–F, 4B–D). Myanmar	*** C. perplexa ***
–	Body larger, 11.5–15.2 mm long, width of midbody metazonae 2.2–3.4 mm. Velum a prominent sac, exomere strongly curved, clearly shorter than endomere, without an outgrowth at base (Fig. 4A–D). Northern Thailand	***C.enghoffi* sp. n.**

## Conclusions

The diplopod diversity in Thailand has hitherto been reported to total 228 species (Likhitrakarn et al. 2017, Srisonchai et al. 2018a, b, c, d, Pimvichai et al. 2018). Given that only a single species, *C.enghoffi* sp. n., of the very large micropolydesmid (= small-bodied) family Pyrgodesmidae has been reported from Thailand, there can be no doubt whatsoever that many more micropolydesmids, including those representing not only the Pyrgodesmidae, but also such taxonomically relatively poorly assessed families as Cryptodesmidae, Opisotretidae, Trichopolydesmidae, and Haplodesmidae still await discovery and description in Thailand and the adjacent countries of Southeast Asia.

## Supplementary Material

XML Treatment for Cryptocorypha

XML Treatment for Cryptocorypha enghoffi
